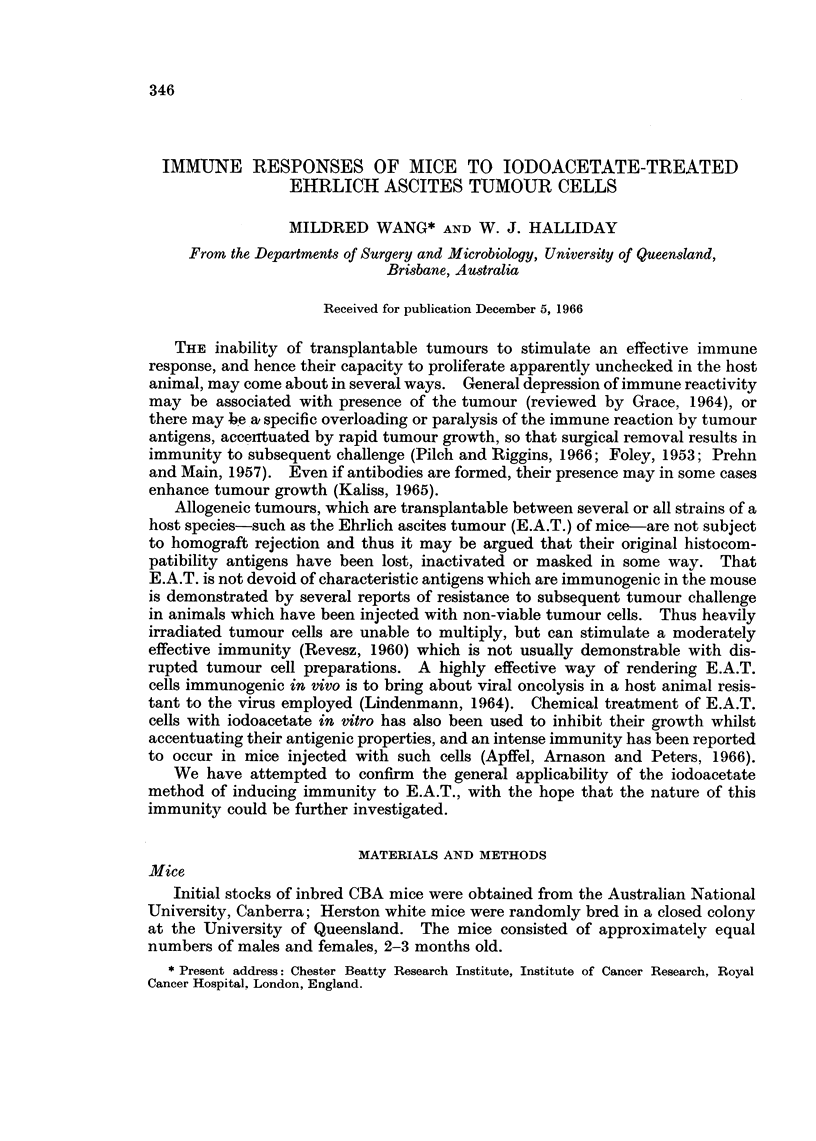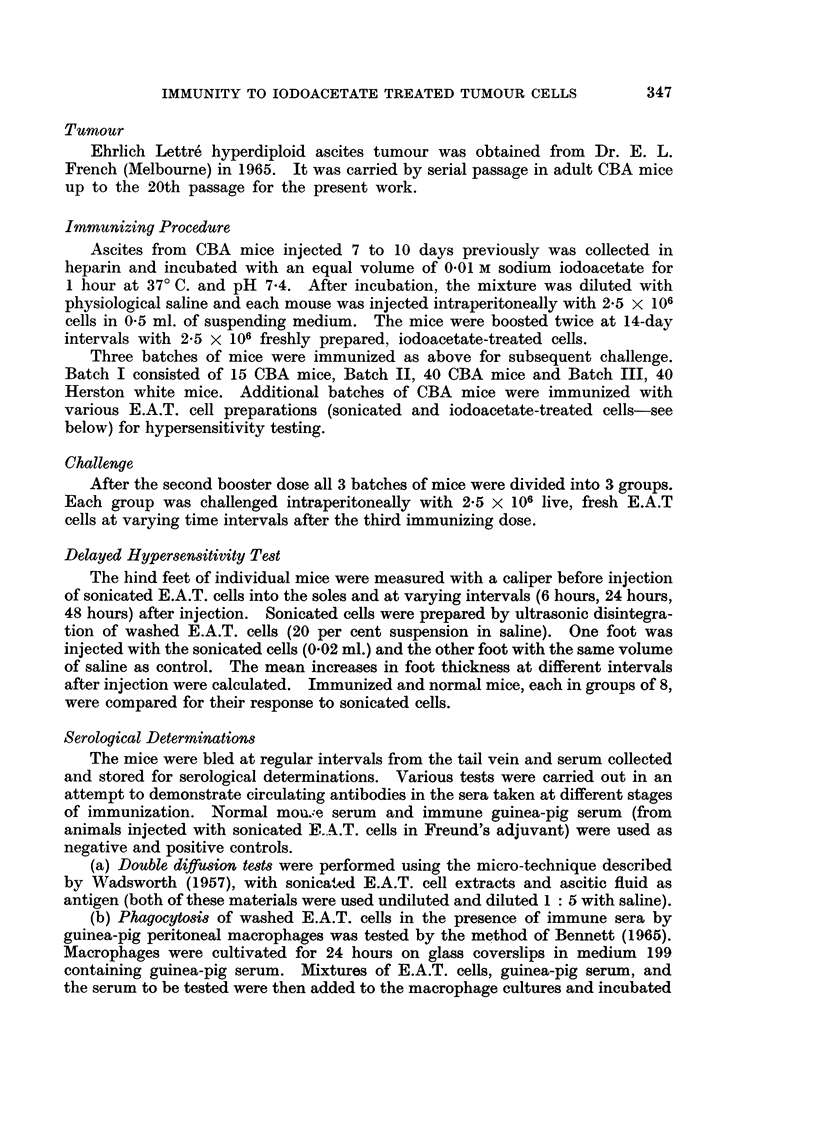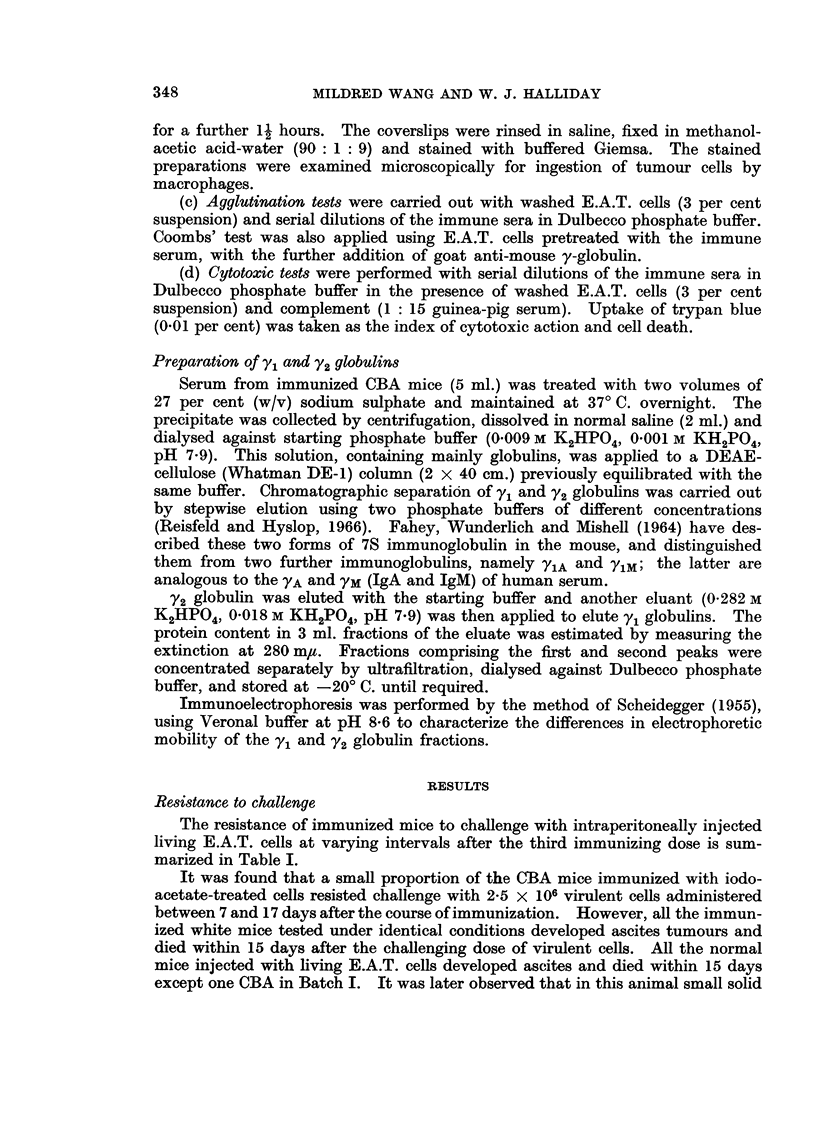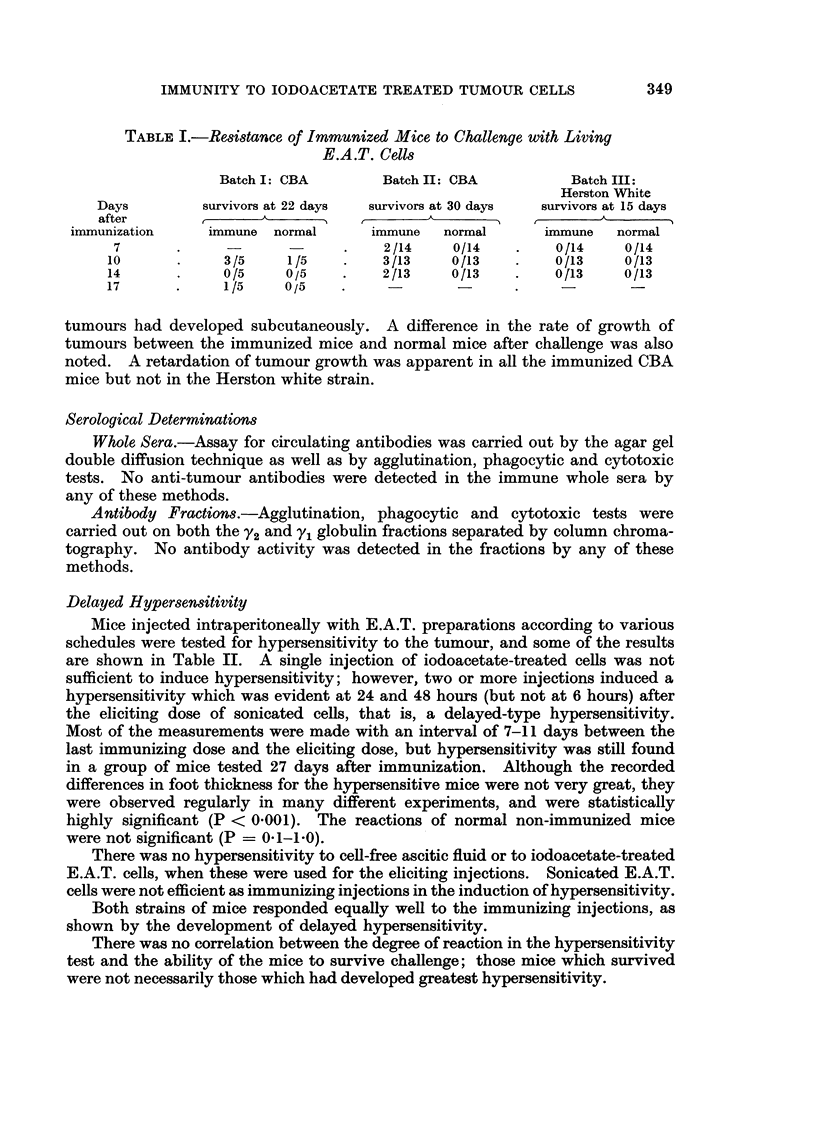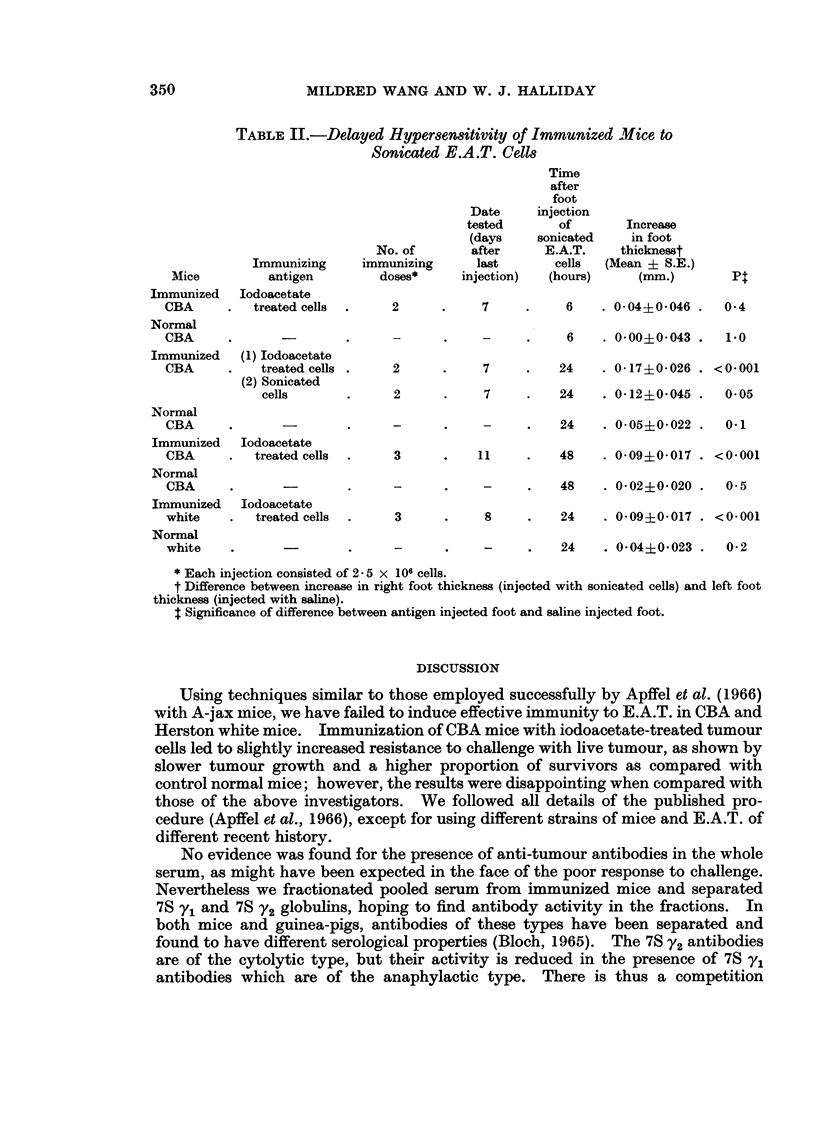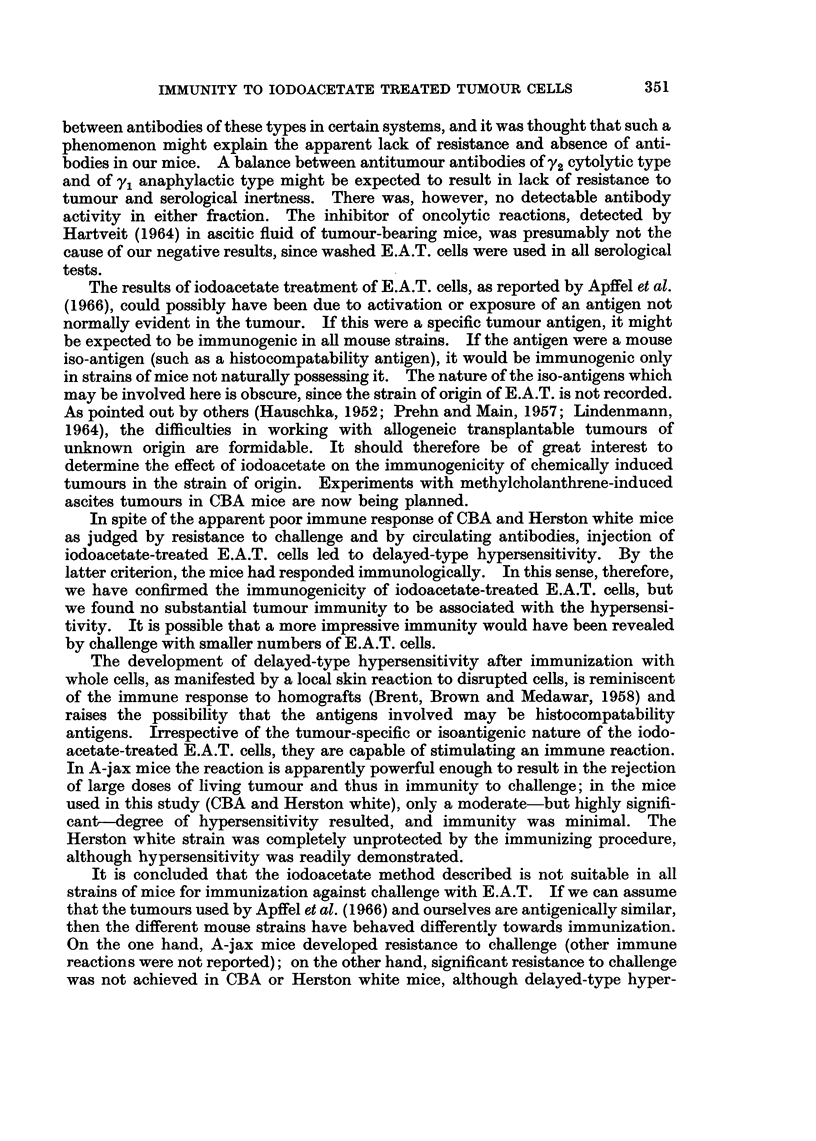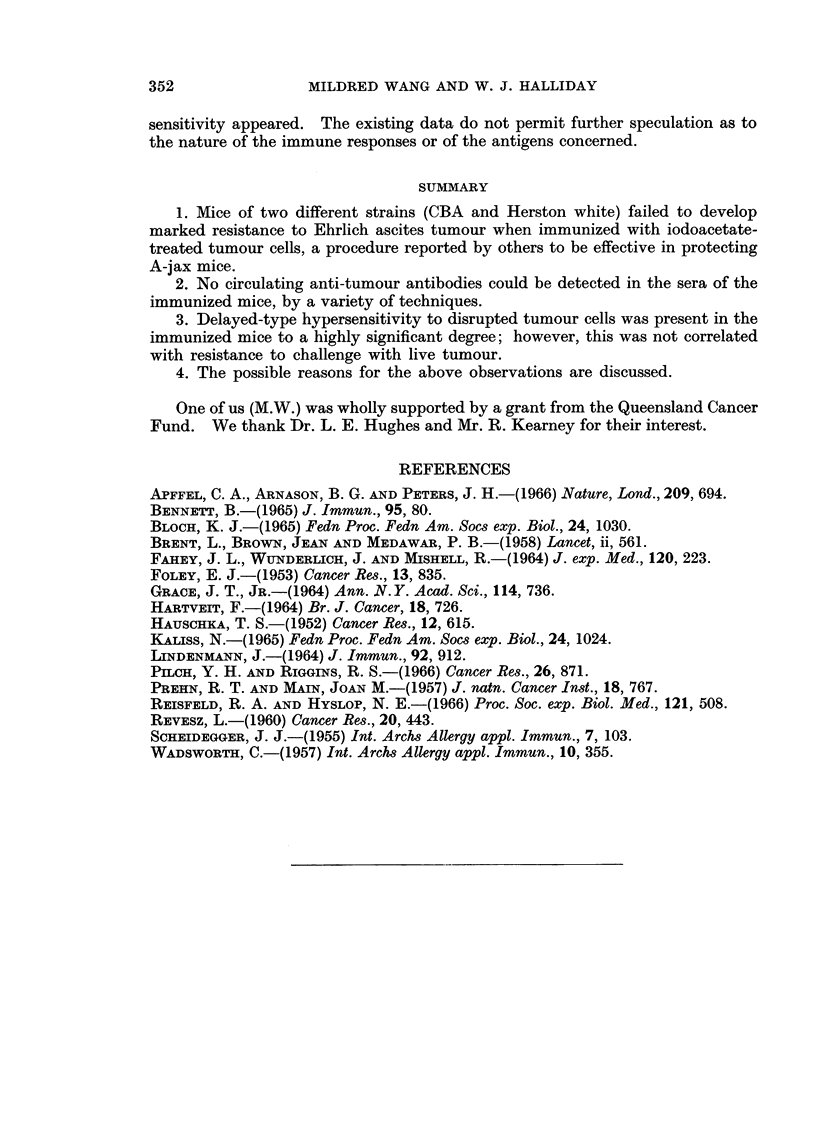# Immune responses of mice to iodoacetate-treated Ehrlich ascites tumour cells.

**DOI:** 10.1038/bjc.1967.39

**Published:** 1967-06

**Authors:** M. Wang, W. J. Halliday


### IMMUNE RESPONSES OF MICE TO IODOACETATE-TREATED EHRLICH ASCITES TUMOUR CELLS

MILDRED WANG* AND W. J. HALLIDAY

*From the Departments of Surgery and Microbiology, University of Queensland, Brisbane, Australia*

Received for publication December 5, 1966

THE inability of transplantable tumours to stimulate an effective immune response, and hence their capacity to proliferate apparently unchecked in the host animal, may come about in several ways. General depression of immune reactivity may be associated with presence of the tumour (reviewed by Grace, 1964), or there may be a specific overloading or paralysis of the immune reaction by tumour antigens, accentuated by rapid tumour growth, so that surgical removal results in immunity to subsequent challenge (Pilch and Riggins, 1966; Foley, 1953; Prehn and Main, 1957). Even if antibodies are formed, their presence may in some cases enhance tumour growth (Kaliss, 1965).

Allogeneic tumours, which are transplantable between several or all strains of a host species—such as the Ehrlich ascites tumour (E.A.T.) of mice—are not subject to homograft rejection and thus it may be argued that their original histocompatibility antigens have been lost, inactivated or masked in some way. That E.A.T. is not devoid of characteristic antigens which are immunogenic in the mouse is demonstrated by several reports of resistance to subsequent tumour challenge in animals which have been injected with non-viable tumour cells. Thus heavily irradiated tumour cells are unable to multiply, but can stimulate a moderately effective immunity (Revesz, 1960) which is not usually demonstrable with disrupted tumour cell preparations. A highly effective way of rendering E.A.T. cells immunogenic *in vivo* is to bring about viral oncolysis in a host animal resistant to the virus employed (Lindenmann, 1964). Chemical treatment of E.A.T. cells with iodoacetate *in vitro* has also been used to inhibit their growth whilst accentuating their antigenic properties, and an intense immunity has been reported to occur in mice injected with such cells (Apffel, Arnason and Peters, 1966).

We have attempted to confirm the general applicability of the iodoacetate method of inducing immunity to E.A.T., with the hope that the nature of this immunity could be further investigated.

### MATERIALS AND METHODS

### *Mice*

Initial stocks of inbred CBA mice were obtained from the Australian National University, Canberra; Herston white mice were randomly bred in a closed colony at the University of Queensland. The mice consisted of approximately equal numbers of males and females, 2–3 months old.

* Present address: Chester Beatty Research Institute, Institute of Cancer Research, Royal Cancer Hospital, London, England.

*Tumour*

Ehrlich Lettré hyperdiploid ascites tumour was obtained from Dr. E. L. French (Melbourne) in 1965. It was carried by serial passage in adult CBA mice up to the 20th passage for the present work.

*Immunizing Procedure*

Ascites from CBA mice injected 7 to 10 days previously was collected in heparin and incubated with an equal volume of 0·01 M sodium iodoacetate for 1 hour at 37° C. and pH 7·4. After incubation, the mixture was diluted with physiological saline and each mouse was injected intraperitoneally with $2 \cdot 5 \times 10^6$ cells in 0·5 ml. of suspending medium. The mice were boosted twice at 14-day intervals with $2 \cdot 5 \times 10^6$ freshly prepared, iodoacetate-treated cells.

Three batches of mice were immunized as above for subsequent challenge. Batch I consisted of 15 CBA mice, Batch II, 40 CBA mice and Batch III, 40 Herston white mice. Additional batches of CBA mice were immunized with various E.A.T. cell preparations (sonicated and iodoacetate-treated cells—see below) for hypersensitivity testing.

*Challenge*

After the second booster dose all 3 batches of mice were divided into 3 groups. Each group was challenged intraperitoneally with $2 \cdot 5 \times 10^6$ live, fresh E.A.T cells at varying time intervals after the third immunizing dose.

*Delayed Hypersensitivity Test*

The hind feet of individual mice were measured with a caliper before injection of sonicated E.A.T. cells into the soles and at varying intervals (6 hours, 24 hours, 48 hours) after injection. Sonicated cells were prepared by ultrasonic disintegration of washed E.A.T. cells (20 per cent suspension in saline). One foot was injected with the sonicated cells (0·02 ml.) and the other foot with the same volume of saline as control. The mean increases in foot thickness at different intervals after injection were calculated. Immunized and normal mice, each in groups of 8, were compared for their response to sonicated cells.

*Serological Determinations*

The mice were bled at regular intervals from the tail vein and serum collected and stored for serological determinations. Various tests were carried out in an attempt to demonstrate circulating antibodies in the sera taken at different stages of immunization. Normal mouse serum and immune guinea-pig serum (from animals injected with sonicated E.A.T. cells in Freund's adjuvant) were used as negative and positive controls.

(a) *Double diffusion tests* were performed using the micro-technique described by Wadsworth (1957), with sonicated E.A.T. cell extracts and ascitic fluid as antigen (both of these materials were used undiluted and diluted 1 : 5 with saline).

(b) *Phagocytosis* of washed E.A.T. cells in the presence of immune sera by guinea-pig peritoneal macrophages was tested by the method of Bennett (1965). Macrophages were cultivated for 24 hours on glass coverslips in medium 199 containing guinea-pig serum. Mixtures of E.A.T. cells, guinea-pig serum, and the serum to be tested were then added to the macrophage cultures and incubated for a further $1\frac{1}{2}$ hours. The coverslips were rinsed in saline, fixed in methanol-acetic acid-water (90 : 1 : 9) and stained with buffered Giemsa. The stained preparations were examined microscopically for ingestion of tumour cells by macrophages.

(c) *Agglutination tests* were carried out with washed E.A.T. cells (3 per cent suspension) and serial dilutions of the immune sera in Dulbecco phosphate buffer. Coombs' test was also applied using E.A.T. cells pretreated with the immune serum, with the further addition of goat anti-mouse $\gamma$-globulin.

(d) *Cytotoxic tests* were performed with serial dilutions of the immune sera in Dulbecco phosphate buffer in the presence of washed E.A.T. cells (3 per cent suspension) and complement (1 : 15 guinea-pig serum). Uptake of trypan blue (0·01 per cent) was taken as the index of cytotoxic action and cell death.

*Preparation of $\gamma_1$ and $\gamma_2$ globulins*

Serum from immunized CBA mice (5 ml.) was treated with two volumes of 27 per cent (w/v) sodium sulphate and maintained at 37° C. overnight. The precipitate was collected by centrifugation, dissolved in normal saline (2 ml.) and dialysed against starting phosphate buffer (0·009 M $K_2HPO_4$, 0·001 M $KH_2PO_4$, pH 7·9). This solution, containing mainly globulins, was applied to a DEAE-cellulose (Whatman DE-1) column (2 × 40 cm.) previously equilibrated with the same buffer. Chromatographic separation of $\gamma_1$ and $\gamma_2$ globulins was carried out by stepwise elution using two phosphate buffers of different concentrations (Reisfeld and Hyslop, 1966). Fahey, Wunderlich and Mishell (1964) have described these two forms of 7S immunoglobulin in the mouse, and distinguished them from two further immunoglobulins, namely $\gamma_{1A}$ and $\gamma_{1M}$; the latter are analogous to the $\gamma_A$ and $\gamma_M$ (IgA and IgM) of human serum.

$\gamma_2$ globulin was eluted with the starting buffer and another eluant (0·282 M $K_2HPO_4$, 0·018 M $KH_2PO_4$, pH 7·9) was then applied to elute $\gamma_1$ globulins. The protein content in 3 ml. fractions of the eluate was estimated by measuring the extinction at 280 m$\mu$. Fractions comprising the first and second peaks were concentrated separately by ultrafiltration, dialysed against Dulbecco phosphate buffer, and stored at −20° C. until required.

Immunoelectrophoresis was performed by the method of Scheidegger (1955), using Veronal buffer at pH 8·6 to characterize the differences in electrophoretic mobility of the $\gamma_1$ and $\gamma_2$ globulin fractions.

### RESULTS

*Resistance to challenge*

The resistance of immunized mice to challenge with intraperitoneally injected living E.A.T. cells at varying intervals after the third immunizing dose is summarized in Table I.

It was found that a small proportion of the CBA mice immunized with iodoacetate-treated cells resisted challenge with 2·5 × $10^6$ virulent cells administered between 7 and 17 days after the course of immunization. However, all the immunized white mice tested under identical conditions developed ascites tumours and died within 15 days after the challenging dose of virulent cells. All the normal mice injected with living E.A.T. cells developed ascites and died within 15 days except one CBA in Batch I. It was later observed that in this animal small solid

TABLE I.—*Resistance of Immunized Mice to Challenge with Living E.A.T. Cells*

| Days after immunization | Batch I: CBA survivors at 22 days immune | normal | Batch II: CBA survivors at 30 days immune | normal | Batch III: Herston White survivors at 15 days immune | normal |
|---|---|---|---|---|---|---|
| 7 | — | — | 2/14 | 0/14 | 0/14 | 0/14 |
| 10 | 3/5 | 1/5 | 3/13 | 0/13 | 0/13 | 0/13 |
| 14 | 0/5 | 0/5 | 2/13 | 0/13 | 0/13 | 0/13 |
| 17 | 1/5 | 0/5 | — | — | — | — |

tumours had developed subcutaneously. A difference in the rate of growth of tumours between the immunized mice and normal mice after challenge was also noted. A retardation of tumour growth was apparent in all the immunized CBA mice but not in the Herston white strain.

*Serological Determinations*

*Whole Sera.*—Assay for circulating antibodies was carried out by the agar gel double diffusion technique as well as by agglutination, phagocytic and cytotoxic tests. No anti-tumour antibodies were detected in the immune whole sera by any of these methods.

*Antibody Fractions.*—Agglutination, phagocytic and cytotoxic tests were carried out on both the $\gamma_2$ and $\gamma_1$ globulin fractions separated by column chromatography. No antibody activity was detected in the fractions by any of these methods.

*Delayed Hypersensitivity*

Mice injected intraperitoneally with E.A.T. preparations according to various schedules were tested for hypersensitivity to the tumour, and some of the results are shown in Table II. A single injection of iodoacetate-treated cells was not sufficient to induce hypersensitivity; however, two or more injections induced a hypersensitivity which was evident at 24 and 48 hours (but not at 6 hours) after the eliciting dose of sonicated cells, that is, a delayed-type hypersensitivity. Most of the measurements were made with an interval of 7–11 days between the last immunizing dose and the eliciting dose, but hypersensitivity was still found in a group of mice tested 27 days after immunization. Although the recorded differences in foot thickness for the hypersensitive mice were not very great, they were observed regularly in many different experiments, and were statistically highly significant ($P < 0{\cdot}001$). The reactions of normal non-immunized mice were not significant ($P = 0{\cdot}1$–$1{\cdot}0$).

There was no hypersensitivity to cell-free ascitic fluid or to iodoacetate-treated E.A.T. cells, when these were used for the eliciting injections. Sonicated E.A.T. cells were not efficient as immunizing injections in the induction of hypersensitivity.

Both strains of mice responded equally well to the immunizing injections, as shown by the development of delayed hypersensitivity.

There was no correlation between the degree of reaction in the hypersensitivity test and the ability of the mice to survive challenge; those mice which survived were not necessarily those which had developed greatest hypersensitivity.

TABLE II.—*Delayed Hypersensitivity of Immunized Mice to Sonicated E.A.T. Cells*

| Mice | Immunizing antigen | No. of immunizing doses* | Date tested (days after last injection) | Time after foot injection of sonicated E.A.T. cells (hours) | Increase in foot thickness† (Mean ± S.E.) (mm.) | P‡ |
|---|---|---|---|---|---|---|
| Immunized CBA | Iodoacetate treated cells | 2 | 7 | 6 | 0·04±0·046 | 0·4 |
| Normal CBA | — | – | – | 6 | 0·00±0·043 | 1·0 |
| Immunized CBA | (1) Iodoacetate treated cells | 2 | 7 | 24 | 0·17±0·026 | <0·001 |
| | (2) Sonicated cells | 2 | 7 | 24 | 0·12±0·045 | 0·05 |
| Normal CBA | — | – | – | 24 | 0·05±0·022 | 0·1 |
| Immunized CBA | Iodoacetate treated cells | 3 | 11 | 48 | 0·09±0·017 | <0·001 |
| Normal CBA | — | – | – | 48 | 0·02±0·020 | 0·5 |
| Immunized white | Iodoacetate treated cells | 3 | 8 | 24 | 0·09±0·017 | <0·001 |
| Normal white | — | – | – | 24 | 0·04±0·023 | 0·2 |

* Each injection consisted of $2 \cdot 5 \times 10^6$ cells.

† Difference between increase in right foot thickness (injected with sonicated cells) and left foot thickness (injected with saline).

‡ Significance of difference between antigen injected foot and saline injected foot.

### DISCUSSION

Using techniques similar to those employed successfully by Apffel *et al.* (1966) with A-jax mice, we have failed to induce effective immunity to E.A.T. in CBA and Herston white mice. Immunization of CBA mice with iodoacetate-treated tumour cells led to slightly increased resistance to challenge with live tumour, as shown by slower tumour growth and a higher proportion of survivors as compared with control normal mice; however, the results were disappointing when compared with those of the above investigators. We followed all details of the published procedure (Apffel *et al.*, 1966), except for using different strains of mice and E.A.T. of different recent history.

No evidence was found for the presence of anti-tumour antibodies in the whole serum, as might have been expected in the face of the poor response to challenge. Nevertheless we fractionated pooled serum from immunized mice and separated 7S $\gamma_1$ and 7S $\gamma_2$ globulins, hoping to find antibody activity in the fractions. In both mice and guinea-pigs, antibodies of these types have been separated and found to have different serological properties (Bloch, 1965). The 7S $\gamma_2$ antibodies are of the cytolytic type, but their activity is reduced in the presence of 7S $\gamma_1$ antibodies which are of the anaphylactic type. There is thus a competition between antibodies of these types in certain systems, and it was thought that such a phenomenon might explain the apparent lack of resistance and absence of antibodies in our mice. A balance between antitumour antibodies of $\gamma_2$ cytolytic type and of $\gamma_1$ anaphylactic type might be expected to result in lack of resistance to tumour and serological inertness. There was, however, no detectable antibody activity in either fraction. The inhibitor of oncolytic reactions, detected by Hartveit (1964) in ascitic fluid of tumour-bearing mice, was presumably not the cause of our negative results, since washed E.A.T. cells were used in all serological tests.

The results of iodoacetate treatment of E.A.T. cells, as reported by Apffel *et al.* (1966), could possibly have been due to activation or exposure of an antigen not normally evident in the tumour. If this were a specific tumour antigen, it might be expected to be immunogenic in all mouse strains. If the antigen were a mouse iso-antigen (such as a histocompatability antigen), it would be immunogenic only in strains of mice not naturally possessing it. The nature of the iso-antigens which may be involved here is obscure, since the strain of origin of E.A.T. is not recorded. As pointed out by others (Hauschka, 1952; Prehn and Main, 1957; Lindenmann, 1964), the difficulties in working with allogeneic transplantable tumours of unknown origin are formidable. It should therefore be of great interest to determine the effect of iodoacetate on the immunogenicity of chemically induced tumours in the strain of origin. Experiments with methylcholanthrene-induced ascites tumours in CBA mice are now being planned.

In spite of the apparent poor immune response of CBA and Herston white mice as judged by resistance to challenge and by circulating antibodies, injection of iodoacetate-treated E.A.T. cells led to delayed-type hypersensitivity. By the latter criterion, the mice had responded immunologically. In this sense, therefore, we have confirmed the immunogenicity of iodoacetate-treated E.A.T. cells, but we found no substantial tumour immunity to be associated with the hypersensitivity. It is possible that a more impressive immunity would have been revealed by challenge with smaller numbers of E.A.T. cells.

The development of delayed-type hypersensitivity after immunization with whole cells, as manifested by a local skin reaction to disrupted cells, is reminiscent of the immune response to homografts (Brent, Brown and Medawar, 1958) and raises the possibility that the antigens involved may be histocompatability antigens. Irrespective of the tumour-specific or isoantigenic nature of the iodoacetate-treated E.A.T. cells, they are capable of stimulating an immune reaction. In A-jax mice the reaction is apparently powerful enough to result in the rejection of large doses of living tumour and thus in immunity to challenge; in the mice used in this study (CBA and Herston white), only a moderate—but highly significant—degree of hypersensitivity resulted, and immunity was minimal. The Herston white strain was completely unprotected by the immunizing procedure, although hypersensitivity was readily demonstrated.

It is concluded that the iodoacetate method described is not suitable in all strains of mice for immunization against challenge with E.A.T. If we can assume that the tumours used by Apffel *et al.* (1966) and ourselves are antigenically similar, then the different mouse strains have behaved differently towards immunization. On the one hand, A-jax mice developed resistance to challenge (other immune reactions were not reported); on the other hand, significant resistance to challenge was not achieved in CBA or Herston white mice, although delayed-type hypersensitivity appeared. The existing data do not permit further speculation as to the nature of the immune responses or of the antigens concerned.

SUMMARY

1. Mice of two different strains (CBA and Herston white) failed to develop marked resistance to Ehrlich ascites tumour when immunized with iodoacetate-treated tumour cells, a procedure reported by others to be effective in protecting A-jax mice.

2. No circulating anti-tumour antibodies could be detected in the sera of the immunized mice, by a variety of techniques.

3. Delayed-type hypersensitivity to disrupted tumour cells was present in the immunized mice to a highly significant degree; however, this was not correlated with resistance to challenge with live tumour.

4. The possible reasons for the above observations are discussed.

One of us (M.W.) was wholly supported by a grant from the Queensland Cancer Fund. We thank Dr. L. E. Hughes and Mr. R. Kearney for their interest.

### REFERENCES

APFFEL, C. A., ARNASON, B. G. AND PETERS, J. H.—(1966) *Nature, Lond.*, **209**, 694.
BENNETT, B.—(1965) *J. Immun.*, **95**, 80.
BLOCH, K. J.—(1965) *Fedn Proc. Fedn Am. Socs exp. Biol.*, **24**, 1030.
BRENT, L., BROWN, JEAN AND MEDAWAR, P. B.—(1958) *Lancet*, ii, 561.
FAHEY, J. L., WUNDERLICH, J. AND MISHELL, R.—(1964) *J. exp. Med.*, **120**, 223.
FOLEY, E. J.—(1953) *Cancer Res.*, **13**, 835.
GRACE, J. T., JR.—(1964) *Ann. N.Y. Acad. Sci.*, **114**, 736.
HARTVEIT, F.—(1964) *Br. J. Cancer*, **18**, 726.
HAUSCHKA, T. S.—(1952) *Cancer Res.*, **12**, 615.
KALISS, N.—(1965) *Fedn Proc. Fedn Am. Socs exp. Biol.*, **24**, 1024.
LINDENMANN, J.—(1964) *J. Immun.*, **92**, 912.
PILCH, Y. H. AND RIGGINS, R. S.—(1966) *Cancer Res.*, **26**, 871.
PREHN, R. T. AND MAIN, JOAN M.—(1957) *J. natn. Cancer Inst.*, **18**, 767.
REISFELD, R. A. AND HYSLOP, N. E.—(1966) *Proc. Soc. exp. Biol. Med.*, **121**, 508.
REVESZ, L.—(1960) *Cancer Res.*, **20**, 443.
SCHEIDEGGER, J. J.—(1955) *Int. Archs Allergy appl. Immun.*, **7**, 103.
WADSWORTH, C.—(1957) *Int. Archs Allergy appl. Immun.*, **10**, 355.